# The association between cardiovascular risk and cardiovascular magnetic resonance measures of fibrosis: the Multi-Ethnic Study of Atherosclerosis (MESA)

**DOI:** 10.1186/s12968-015-0121-5

**Published:** 2015-02-12

**Authors:** Colin J Yi, Colin O Wu, Michael Tee, Chia-Ying Liu, Gustavo J Volpe, Martin R Prince, Gregory W Hundley, Antoinette S Gomes, Rob J van der Geest, Susan Heckbert, João A Lima, David A Bluemke

**Affiliations:** National Institutes of Health, Radiology and Imaging Sciences, Bethesda, MD USA; Department of Medicine, Johns Hopkins University, Baltimore, MD USA; Cornell University, Cornell MRI Facility, Ithaca, NY USA; Wake Forest University, Health Sciences, Winston-Salem, NC USA; UCLA, School of Medicine, Los Angeles, CA USA; Department of Radiology, Leiden University Medical Center, Leiden, The Netherlands; University of Washington, Cardiovascular Health Research Unit, Seattle, WA USA; National Heart, Lung and Blood Institute, Office of Biostatistics Research, Bethesda, MD USA

**Keywords:** Myocardium, Cardiovascular magnetic resonance, Risk factors

## Abstract

**Background:**

Risk scores for cardiovascular disease (CVD) are in common use to integrate multiple cardiovascular risk factors in order to identify individuals at greatest risk for disease. The purpose of this study was to determine if individuals at greater cardiovascular risk have T_1_ mapping indices by cardiovascular magnetic resonance (CMR) indicative of greater myocardial fibrosis.

**Methods:**

CVD risk scores for 1208 subjects (men, 50.8%) ages 55–94 years old were evaluated in the Multiethnic Study of Atherosclerosis (MESA) at six centers. T_1_ times were determined at 1.5Tesla before and after gadolinium administration (0.15 mmol/kg) using a modified Look-Locker pulse sequence. The relationship between CMR measures (native T_1_, 12 and 25 minute post-gadolinium T_1_, partition coefficient and extracellular volume fraction) and 14 established different cardiovascular risk scores were determined using regression analysis. Bootstrapping analysis with analysis of variance was used to compare different CMR measures. CVD risk scores were significantly different for men and women (p < 0.001).

**Results:**

25 minute post gadolinium T_1_ time showed more statistically significant associations with risk scores (10/14 scores, 71%) compared to other CMR indices (e.g. native T_1_ (7/14 scores, 50%) and partition coefficient (7/14, 50%) in men. Risk scores, particularly the new 2013 AHA/ASCVD risk score, did not correlate with any CMR fibrosis index.

**Conclusions:**

Men with greater CVD risk had greater CMR indices of myocardial fibrosis. T_1_ times at greater delay time (25 minutes) showed better agreement with commonly used risk score indices compared to ECV and native T_1_ time.

**Clinical trial registration:**

http://www.mesa-nhlbi.org/, NCT00005487.

## Background

Overt cardiovascular disease (CVD) encompasses a wide array of late stage outcomes (e.g., coronary heart disease, heart failure, myocardial infarction) that are expensive to treat and that may be preventable. In order to better manage cardiovascular health, CVD risk models have been developed to relate an individual’s risk for adverse CVD outcomes with various biomarkers [1,2]. The Framingham Study pioneered the translation of common risk factors of CVD into an overall quantitative assessment of risk score [3]. Risk score models such as Framingham include various combinations of age, gender, blood pressure, etc. using different weighting coefficients for each risk parameter. High risk individuals may be subject to more intensive assessment and therapy [4]. Recent efforts to further improve primary CVD prevention have led to the development of the new 2013 AHA guidelines [5]. Imaging is increasingly used to phenotype patients. For example, the relationship of risk scores to computed tomography (CT) calcium score has been previously studied [6].

The myocardium responds to physiological and pathological stress by remodeling with increased deposition of interstitial collagen [7]. The presence of diffuse myocardial fibrosis has been known to confer risk for various types of CVD [8], and common traditional cardiovascular risk factors are known to be associated with myocardial fibrosis [9-12]. One noninvasive method to assess diffuse myocardial fibrosis is by measuring the T_1_ relaxivity of myocardial tissue before and/or after administration of a gadolinium based contrast agent using cardiovascular magnetic resonance (CMR) [13,14]. The rate of contrast relaxation is dependent on the inherent tissue properties of the myocardium, thus allowing characterization of myocardial composition. Extracellular volume (ECV) is an additional index of fibrosis that is related to the ratio of interstitial space to total myocardial volume [15]. T_1_ and ECV indices of myocardial fibrosis have been validated in select patient populations [16,17].

CMR indices of myocardial fibrosis are relatively new biomarkers and their applicability in a general, low to moderate risk population has not yet been determined. The Multi-Ethnic Study of Atherosclerosis (MESA) study provides the opportunity to explore the validity of CMR indices of myocardial fibrosis with respect to risk for CVD in a largely asymptomatic subject pool. The purpose of this study was to determine the relationship between CMR fibrosis indices and CVD risk score models in a large, population-based study. We hypothesize CVD risk scores will be correlated with CMR fibrosis indices.

## Methods

Details of the design and organization of the Multi-Ethnic Study of Atherosclerosis (MESA) trial have been reported previously [18]. From 2000–2002, 6814 men and women ranging from ages of 45 to 84 years were enrolled in the baseline MESA study. MESA participants were community-dwelling men and women, of African American, Hispanic, white and Chinese American descent. Study subjects underwent continued follow up for at periodic intervals, and CMR was performed at the 5^th^ follow-up examination from 2010–2012 (designated as the “MESA 5” follow-up examination). Institutional review board approval was obtained and all subjects signed informed consent for MESA procedures.

Smoking was defined as never, former (smoked ≥ 100 cigarettes in lifetime), or current (smoked cigarettes in last 30 days). Hypertension was defined as systolic blood pressure ≥ 140 mm Hg, diastolic blood pressure ≥ 90 mm Hg, self-reported hypertension or use of antihypertensive medication. Hypertrophy of the left ventricle was defined as “no” (absence of LV hypertrophy) or “yes” (presence of LV hypertrophy). Type II diabetes was defined as fasting glucose > 125 mg/dl or use of diabetic medication.

### Lipid, lipoprotein, and other laboratory assays

Blood was drawn after a 12 hour fast, and samples were stored at −70°C. Lipids, insulin, and glucose were measured at a central laboratory (Collaborative Studies Clinical Laboratory at Fairview University Medical Center, Minneapolis, Minnesota). Lipids were assayed on thawed EDTA plasma within 2 weeks of sample collection, using Centers for Disease Control Prevention/NHLBI standards. High-density lipid cholesterol (HDL-C) was measured using the cholesterol oxidase method (Roche Diagnostics, Indianapolis, Indiana) after precipitation of non-HDL-C with magnesium/dextran (coefficient of variation 2.9%). LDL-C was calculated using Friedewald equation [19]. The serum concentration of NT-proBNP was measured using a highly sensitive and specific immunoassay based on a double-antibody sandwich technique (Roche Diagnostics Corporation, Indianapolis, IN, USA) [20].

### CMR for evaluation of diffuse fibrosis

Study subjects with prior myocardial infarction or focal late gadolinium enhancement were excluded from analysis. The CMR and T_1_ mapping protocol in MESA has been described [21]. Pre-contrast short axis (Modified Look Locker Inversion Recovery) MOLLI imaging at the mid ventricle was obtained followed by post-contrast image acquisition 12 and 25 min after contrast injection. The MOLLI sequence acquired a set of 11 source images over 17 heartbeats. An inversion recovery echo triggered sequence consisted of 3 inversion pulses at the following inversion times: 100, 200, and 350 ms. Additional scanning parameters were summarized as follows: flip angle = 35°; repetition time = 2.2 ms; echo time = 1.1 ms; field of view = 360 × 360 mm; matrix = 192 × 183; slice thickness = 8 mm; generalized autocalibrating partially parallel acquisitions factor = 2.

MASS research software (Department of Radiology, Leiden University Medical Center, Leiden, and the Netherlands) was used to create T_1_ maps. Levenberg-Marquardt algorithm allowed a 3 parameter curve fit using MOLLI as a source image to extract T_1_ time for each pixel. Myocardial T_1_ time was then determined by careful drawing of the region of interest to encompass the myocardium tissue only, so that the trabeculation, blood pool and epicardial fat were excluded. The partition coefficient (λ) was calculated by using a 3 point linear fit to determine the resulting slope (ΔT_1myo_/ΔT_1blood_). Extracellular volume (ECV) as a percentage was evaluated by multiplying the partition coefficient by (1 – hematocrit). Expected associations with greater degree of myocardial fibrosis by CMR are higher ECV and native T1 time, but lower post gadolinium T_1_ time [15-17].

### Risk score models

Risk scores were based on prior systematic and blinded review by Allan et al. [22], including Edinburgh (CVD), Edinburgh (CHD), BNF, ASSIGN, PROCAM, Framingham, National Cholesterol Education Program, and Reynolds Risk Score (Table 1). In addition, Framingham and 5 other risk scores (MI risk score, stroke risk score, death from CHD and CVD risk scores, MESA derived risk score, and the new AHA/ASCVD risk score) were available for each MESA 5 subject [5,23,24]. The endpoints used for each of the risk scores are shown in Table 1.

**Table 1:** **Endpoint prediction for each of 14 risk scores**

Risk scores*	**Endpoint prediction**	**Risk factors included in model** ^**‡**^
Edinburgh (CHD)^1^	10 year CHD	Age, Gender, Smoker, Diabetes, LVH, SBP, Chol, HDL
Edinburgh (CVD)^1^	10 year CVD	Age, Gender, Smoker, Diabetes, LVH, SBP, Chol, HDL
Death (CHD)^1^	10 year death from CHD	Age, Gender, Smoker, Diabetes, LVH, SBP, Chol, HDL
Death (CVD)^1^	10 year death from CVD	Age, Gender, Smoker, Diabetes, LVH, SBP, Chol, HDL
Myocardial Infarction (MI)^1^	10 year MI	Age, Gender, Smoker, Diabetes, LVH, SBP, Chol, HDL
Stroke^1^	10 year stroke	Age, Gender, Smoker, Diabetes, LVH, SBP, Chol, HDL
Framingham (CVD)^3^	10 year CVD	Age, Gender, Smoker, SBP, Chol, HDL, HTN Med
National Cholesterol Education Program (NCEP)^4^	10 year CHD	Age, Smoker, Diabetes, SBP, HDL, LDL
British National Formulary (BNF)^1^	10 year CVD	Age, Gender, Smoker, Diabetes, LVH, SBP, Chol, HDL
ASSIGN^1^	10 year CVD	Age, Gender, Smoker, Diabetes, SBP, Chol, HDL, Cigarettes, MI Fam
PROCAM^2^	10 year CHD	Diabetes, SBP, HDL, LDL, Trig, Glucose, HTN Med
Reynolds^5^	10 year CHD	Age, Gender, Smoker, Diabetes, SBP, Chol, HDL, CRP, Hemoglobin, MI Fam
MESA^6^	5 year HF	Age, Gender, Smoker, BMI Grade, SBP, Heart Rate, Diabetic Status, BNP, LV Mass
Atherosclerotic Cardiovascular Disease (ASCVD)^7^	10 year CVD	Age, Gender, Smoker, Diabetes, SBP, Chol, HDL

*References indicated below.

CVD – Cardiovascular Disease; CHD – Coronary Heart Disease; HF – Heart Failure; Chol – Total Cholesterol; LVH – Left Ventricular Hypertrophy; SBP – Systolic Blood Pressure; HTN Med – Hypertension Medication; MI Fam – Family History of Myocardial Infarction; BNP – proBNP levels; LV Mass – Left Ventricular Mass; Trig – triglycerides; BMI – body mass index; CRP – c reactive protein.

^‡^Risk Scores were calculated as in [25].

^1-7^See Appendix for Risk score references.

### Statistical analysis

All analyses were stratified by gender to reduce the effects of potential confounders. Risk scores were categorized as a) “low”, “intermediate” and “high”, or b) “low”, “average”, “intermediate” and “high” depending on the conventions of each score [26]. However, for evaluation of association between each of the 14 risk scores and T_1_ times or ECV, the risk scores were treated as continuous variables. Correlations between each risk score and T_1_ times or ECV were tested using linear regression models adjusted for heart rate. T_1_ times were studied at both 12 and 25 minutes after gadolinium administration. The Generalized Additive Model (GAM) was employed to test for departures from the linear regression models. If the resulting model suggested non-linearity, the corresponding data points were refitted using a linear spline regression model. Bootstrapping analysis was performed with 10,000 iterations to test for reliability of correlation results [27].

## Results

Complete CMR data as well as clinical and serologic data were available (Table 2) for 1231 subjects (625 (50.8%) women and 606 (49.2%) men). Characteristics of the MESA 5 population are shown in Table 2. Age and BMI were not significantly different between men and women. The racial composition included Caucasians (51.7%), African Americans (22.5%), Hispanic (14.1%), and Chinese Americans (11.7%). 6.8% of study subjects were current smokers. The prevalence of hypertension and diabetes was 52.1% and 15%, respectively. All subjects were free from prior myocardial infarction or CVD event at the time of MESA exam 5.

**Table 2:** **Characteristics of study population**

**Demographics**	**Women (n = 625)**	**Men (n = 606)**	**p-value**
Age (yrs)	67 ± 9	67 ± 9	0.94
Height (cm)	160 ± 6.6	174 ± 7.5	<0.001
Weight (lb)	161 ± 36	188 ± 35	<0.001
BMI (kg/m2)	28 ± 6	28 ± 5	0.24
White/African/Chinese/Hispanic (%)	54/23/11/12	50/22/12/16	0.13
Heart rate (beats/min)	65.3 ± 9.5	63.8 ± 10	0.005
Systolic blood pressure (mm Hg)	122 ± 20	121 ± 18	0.49
Diastolic blood pressure (mm Hg)	65 ± 9	71 ± 9	<0.001
Current smokers	41 (6.6)	43 (7.1)	<0.001
Hypertension	343 (54.9)	298 (49.2)	0.045
Diabetes	87 (13.9)	98 (16.2)	0.002
Metabolic syndrome^‡^	233 (37.3)	182 (30)	0.001
HDL cholesterol (mg/dl)	60.2 ± 16.9	49.1 ± 13.1	<0.001
LDL cholesterol (mg/dl)	110.8 ± 31.2	100.6 ± 30.4	<0.001
Total cholesterol (mg/dl)	193.2 ± 34.6	171.5 ± 34	<0.001
Triglycerides (mg/dl)	111.8 ± 58.7	110 ± 69	0.78
eGFR (ml/min/1.73 m^2^)	85 ± 21	85 ± 17	0.52
Framingham risk score	0.06 ± 0.04	0.13 ± 0.07	<0.001
Native (Pre-contrast) myocardial T_1_ (ms)	986 ± 45	968 ± 38	<0.001
Post-contrast myocardial T_1_ (ms)^§^	505 ± 41	535 ± 34	<0.001
Hematocrit (%)	38.4 ± 2.9	41.6 ± 3.5	<0.001
Extracellular volume fraction (%)^ǁ^	28.1 ± 2.8	25.8 ± 2.9	<0.001

*Values are mean ± SD, %, or n (%).

BMI = body mass index; eGFR = estimated glomerular filtration rate; HDL = high-density lipoprotein, LDL = low-density lipoprotein.

^‡^Defined according to National Cholesterol Education Program guidelines.

^§^25-min post-contrast T1 time.

^ǁ^n = 321 for women; n = 287 for men.

**Figure 1:**
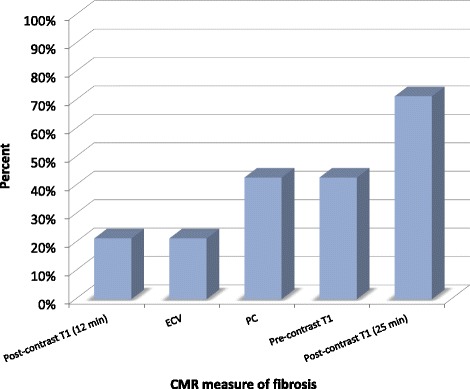
**Percentage of cardiovascular risk models showing significant relationship with CMR indices of fibrosis.** Only risk models that show expected directions of beta coefficients are shown (i.e. increased risk vs. lower post gadolinium T1 times or greater ECV, partition coefficient and native T1 time). For men, 25 minute post gadolinium T1 values showed the most consistent relationship to cardiovascular risk scores. Only models for men are shown. ECV = extracellular volume fraction; λ = partition coefficient. Bars indicate standard deviation.

As expected, CVD risk scores were generally greater for men than women (Table 3). Tables 4, 5, 6, 7 and 8 present the associations between the 14 risk scores and CMR measures of fibrosis, including 12 and 25 min post gadolinium T1 time, native T1, partition coefficient and ECV. Table 5 shows that that 10 of 14 (71%) risk score models (Edinburgh CVD and CHD, MI, stroke, CVD and CHD Death, BNF, Reynolds, MESA and CVD Framingham) showed greater risk score associated with lower post-gadolinium T1 time at 25 minutes in men. The partition coefficient and native T1 times were each associated with 7 risk scores in men (although 1 case of opposite than expected correlation for each). ECV and 12 min T1 time were each associated with 3 risk scores in men occurring in the expected direction (i.e., greater cardiovascular risk correlated with greater fibrosis by CMR). The overall agreement between risk scores and CMR indices is shown in Figure 1.

**Table 3:** Cardiovascular risk score (%) for outcomes* for men and women

**Risk score**	**Women**	**Men**	**p-value**
Edinburgh (CHD)	5.772 ( 4.275 )	12.786 ( 7.034 )	**<0.001**
-	552/51/12	226/300/72	--
Edinburgh (CVD)	11.629 ( 8.233 )	19.339 ( 10.1 )	**<0.001**
-	337/197/81	98/253/247	--
Death (CHD)	1.204 ( 1.936 )	3.455 ( 3.159 )	**<0.001**
-	610/5/0	571/25/2	--
Death (CVD)	3.481 ( 5.027 )	6.398 ( 6.583 )	**<0.001**
-	563/41/11	477/97/24	--
MI	1.819 ( 2.282 )	5.944 ( 4.34 )	**<0.001**
-	605/10/0	512/80/6	--
STROKE	3.017 ( 3.553 )	3.614 ( 3.542 )	**0.003**
-	590/20/5	565/29/4	--
Framingham (CVD)	10.696 ( 8.223 )	25.221 ( 13.78 )	**<0.001**
-	378/167/72	62/184/356	--
NCEP	10.583 ( 6.144 )	11.088 ( 6.365 )	0.162
-	349/212/50	328/210/55	--
BNF	8.789 ( 6.874 )	16.4 ( 9.959 )	**<0.001**
-	446/134/35	150/300/148	--
ASSIGN	13.222 ( 30.631 )	88.814 ( 29.171 )	**<0.001**
-	264/13/96	36/9/516	--
PROCAM	0.407 ( 0.608 )	0.476 ( 0.514 )	**0.033**
-	613/0/0	599/0/0	--
Reynolds	3.711 ( 4.837 )	12.377 ( 9.688 )	**<0.001**
-	549/26/9	293/177/89	--
MESA	2.888 ( 6.518 )	2.854 ( 6.758 )	0.935
-	480 / 14 / 13	471/19/10	--
ASCVD	10.116 ( 9.8 )	14.61 ( 8.895 )	**<0.001**
-	258/111/49	138/159/82	--

*Risk scores and outcomes are defined in Table 1.

Bold indicates p < 0.05.

Below each risk score shows number of subjects categorized as “low”/“medium”/“high” risk.

**Table 4:** **Regression coefficients (β) between cardiovascular risk score and 12 minute post gadolinium T**_**1**_ **time**

**Risk score**	**β (msec/%) women/men** ^**†**^^**‡**^	**CI (95%) women/men** ^**†**^	**p-value women/men** ^*†^
Edinburgh (CHD)	−0.343 / -0.288	[ −1.116 to 0.43 ] / [ −0.668 to 0.093 ]	0.383 / 0.138
Edinburgh (CVD)	−0.154 / -0.244	[ −0.556 to 0.248 ] / [ −0.509 to 0.021 ]	0.453 / 0.071
DEATH (CHD)	−0.011 / -0.701	[ −1.718 to 1.697 ] / [ −1.549 to 0.147 ]	0.99 / 0.105
DEATH (CVD)	0.083 / -0.5	[ −0.574 to 0.741 ] / [ −0.906 to −0.094 ]	0.804 / **0.016**
MI	−0.67 / -0.453	[ −2.12 to 0.78 ] / [ −1.072 to 0.165 ]	0.365 / 0.151
STROKE	−0.054 / -0.541	[ −0.984 to 0.876 ] / [ −1.298 to 0.215 ]	0.909 / 0.16
Framingham (CVD)	−0.119 / -0.206	[ −0.522 to 0.284 ] / [ −0.398 to −0.013 ]	0.562 / **0.037**
NCEP	−0.045 / -0.058	[ −0.587 to 0.496 ] / [ −0.484 to 0.368 ]	0.869 / 0.789
BNF	−0.147 / -0.212	[ −0.628 to 0.334 ] / [ −0.481 to 0.057 ]	0.548 / 0.122
ASSIGN	−0.038 / -0.006	[ −0.146 to 0.07 ] / [ −0.098 to 0.087 ]	0.492 / 0.907
PROCAM	−2.578 / -2.131	[ −8.041 to 2.885 ] / [ −7.328 to 3.067 ]	0.354 / 0.421
Reynolds	−0.504 / -0.339	[ −1.187 to 0.179 ] / [ −0.617 to −0.061 ]	0.148 / **0.017**
MESA	0.126 / -0.391	[ −0.437 to 0.689 ] / [ −0.832 to 0.05 ]	0.661 / 0.082
ASCVD	−0.102 / -0.138	[ −0.505 to 0.301 ] / [ −0.497 to 0.22 ]	0.62 / 0.449

*Bold indicates p < 0.05.

^†^Data represented as regression coefficient for women/men.

^‡^β = regression coefficient expressed as ms/per unit % change in risk score.

**Table 5:** **Regression coefficients (β) between cardiovascular risk score and 25 minute post gadolinium T**_**1**_ **time**

**Risk score**	**β (msec/%) women/men** ^**†**^^**‡**^	**CI (95%) women/men** ^**†**^	**p-value women/men** ^*†^
Edinburgh (CHD)	−0.175 / -0.485	[ −0.939 to 0.589 ] / [ −0.88 to −0.089 ]	0.654 / **0.016**
Edinburgh (CVD)	−0.169 / -0.412	[ −0.566 to 0.229 ] / [ −0.687 to −0.136 ]	0.405 / **0.003**
DEATH (CHD)	−0.44/-1.275	[ −2.126 to 1.246 ] / [ −2.156 to −0.395 ]	0.608 / **0.005**
DEATH (CVD)	−0.215 / -0.798	[ −0.864 to 0.434 ] / [ −1.218 to −0.378 ]	0.515 / **<0.001**
MI	−0.12 / -0.783	[ −1.553 to 1.313 ] / [ −1.427 to −0.14 ]	0.87 / **0.017**
STROKE	−0.27 / -1.067	[ −1.188 to 0.649 ] / [ −1.854 to −0.28 ]	0.564 / **0.008**
Framingham (CVD)	−0.173 / -0.342	[ −0.572 to 0.225 ] / [ −0.542 to −0.142 ]	0.393 / **0.001**
NCEP	0.123 / -0.362	[ −0.414 to 0.659 ] / [ −0.807 to 0.083 ]	0.654 / 0.111
BNF	−0.14 / -0.377	[ −0.615 to 0.335 ] / [ −0.656 to −0.098 ]	0.564 / **0.008**
ASSIGN	−0.023 / 0.026	[ −0.129 to 0.083 ] / [ −0.07 to 0.122 ]	0.666 / 0.598
PROCAM	−0.058 / -2.441	[ −5.48 to 5.365 ] / [ −7.861 to 2.979 ]	0.983 / 0.377
Reynolds	−0.335 / -0.554	[ −1.008 to 0.339 ] / [ −0.839 to −0.268 ]	0.329 / **<0.001**
MESA	0.004 / -0.716	[ −0.562 to 0.57 ] / [ −1.241 to −0.191 ]	0.99 / **0.008**
ASCVD	−0.099 / -0.307	[ −0.492 to 0.293 ] / [ −0.694 to 0.08 ]	0.619 / 0.12

*Bold indicates p < 0.05.

^†^Data represented as regression coefficient for women/men.

^‡^β = regression coefficient expressed as ms/per unit % change in risk score.

**Table 6:** **Regression coefficients (β) between cardiovascular risk score and native T**_**1**_ **time**

**Risk score**	**β (msec/%) women/men** ^**†**^^**‡**^	**CI (95%) women/men** ^**†**^	**p-value women/men** ^*†^
Edinburgh (CHD)	0.248 / 0.118	[ −0.588 to 1.085 ] / [ −0.318 to 0.553 ]	0.56 / 0.596
Edinburgh (CVD)	0.286 / 0.32	[ −0.148 to 0.72 ] / [ 0.017 to 0.623 ]	0.196 / **0.038**
DEATH (CHD)	0.618 / 0.611	[ −1.229 to 2.464 ] / [ −0.359 to 1.582 ]	0.511 / 0.216
DEATH (CVD)	0.268 / 0.565	[ −0.443 to 0.979 ] / [ 0.101 to 1.029 ]	0.459 / **0.017**
MI	1.198 / 0.241	[ −0.368 to 2.765 ] / [ −0.467 to 0.949 ]	0.134 / 0.504
STROKE	0.578 / 1.345	[ −0.428 to 1.583 ] / [ 0.485 to 2.205 ]	0.26 / **0.002**
Framingham (CVD)	0.374 / 0.284	[ −0.061 to 0.809 ] / [ 0.062 to 0.505 ]	0.092 / **0.012**
NCEP	0.457 / 0.12	[ −0.128 to 1.041 ] / [ −0.366 to 0.606 ]	0.126 / 0.628
BNF	0.25 / 0.229	[ −0.269 to 0.77 ] / [ −0.079 to 0.536 ]	0.344 / 0.145
ASSIGN	−0.039 / -0.169	[ −0.159 to 0.081 ] / [ −0.275 to −0.062 ]	0.523 / **0.002**
PROCAM	5.3 / -3.063	[ −0.604 to 11.204 ] / [ −9.027 to 2.9 ]	0.078 / 0.313
Reynolds	0.252 / 0.495	[ −0.51 to 1.015 ] / [ 0.172 to 0.819 ]	0.516 / **0.003**
MESA	0.238 / 0.398	[ −0.357 to 0.833 ] / [ −0.112 to 0.908 ]	0.433 / 0.126
ASCVD	0.302 / 0.423	[ −0.141 to 0.744 ] / [ 0.006 to 0.84 ]	0.181 / **0.047**

*Bold indicates p < 0.05.

^†^Data represented as regression coefficient for women/men.

^‡^β = regression coefficient expressed as ms/per unit % change in risk score.

**Table 7:** **Regression coefficients (β) between cardiovascular risk score and partition coefficient (**λ**)**

**Risk score**	**β (msec/%) women/men** ^**†**^^**‡**^	**CI (95%) women/men** ^**†**^	**p-value women/men** ^*†^
Edinburgh (CHD)	−0.00053 / 0.00001	[ −0.00129 to 0.00023 ] / [ −0.00046 to 0.00047 ]	0.168 / 0.980
Edinburgh (CVD)	−0.00006 / 0.00029	[ −0.00045 to 0.00033 ] / [ −3e-05 to 0.00061 ]	0.764 / 0.080
DEATH (CHD)	−0.00049 / 0.00049	[ −0.00216 to 0.00118 ] / [ −0.00054 to 0.00152 ]	0.566 / 0.350
DEATH (CVD)	−0.00019 / 0.0007	[ −0.00083 to 0.00046 ] / [ 0.00021 to 0.00119 ]	0.568 / **0.005**
MI	0.00031 / 0.00004	[ −0.00111 to 0.00173 ] / [ −0.00071 to 8e-04 ]	0.664 / 0.908
STROKE	−0.00024 / 0.00098	[ −0.00115 to 0.00067 ] / [ 6e-05 to 0.00189 ]	0.608 / **0.037**
Framingham (CVD)	−0.00011 / 0.00032	[ −5e-04 to 0.00028 ] / [ 8e-05 to 0.00055 ]	0.579 / **0.008**
NCEP	−0.00003 / -0.00025	[ −0.00056 to 5e-04 ] / [ −0.00077 to 0.00027 ]	0.924 / 0.348
BNF	−0.00027 / 0.00013	[ −0.00074 to 2e-04 ] / [ −2e-04 to 0.00045 ]	0.262 / 0.448
ASSIGN	−0.00007 / -0.00013	[ −0.00018 to 4e-05 ] / [ −0.00024 to -2e-05 ]	0.214 / **0.024**
PROCAM	0.00324 / -0.00428	[ −0.0021 to 0.00858 ] / [ −0.01063 to 0.00208 ]	0.234 / 0.187
Reynolds	−0.00016 / 0.00047	[ −0.00085 to 0.00053 ] / [ 0.00014 to 8e-04 ]	0.645 / **0.006**
MESA	−0.00007 / 0.00067	[ −0.00061 to 0.00047 ] / [ 0.00014 to 0.00121 ]	0.799 / **0.013**
ASCVD	−0.00002 / 0.00048	[ −0.00044 to 4e-04 ] / [ 3e-05 to 0.00093 ]	0.914 / **0.035**

*Bold indicates p < 0.05.

^†^Data represented as regression coefficient for women/men.

^‡^β = regression coefficient expressed as ms/per unit % change in risk score.

**Table 8:** **Regression coefficients (β) between cardiovascular risk score and extracellular volume fraction (ECV)**

**Risk score**	**β (msec/%) women/men** ^**†**^^**‡**^	**CI (95%) women/men** ^**†**^	**p-value women/men** ^*†^
Edinburgh (CHD)	−0.072 / -0.012	[ −0.142 to −0.002 ] / [ −0.059 to 0.035 ]	**0.043** / 0.612
Edinburgh (CVD)	−0.013 / 0.014	[ −0.051 to 0.025 ] / [ −0.019 to 0.047 ]	0.497 / 0.397
DEATH (CHD)	−0.035 / 0.013	[ −0.2 to 0.131 ] / [ −0.09 to 0.116 ]	0.68 / 0.806
DEATH (CVD)	0.004 / 0.056	[ −0.053 to 0.062 ] / [ 0.007 to 0.105 ]	0.88 / **0.025**
MI	−0.05 / -0.023	[ −0.199 to 0.098 ] / [ −0.097 to 0.05 ]	0.506 / 0.532
STROKE	−0.012 / 0.086	[ −0.106 to 0.082 ] / [ −0.007 to 0.178 ]	0.805 / 0.069
Framingham (CVD)	−0.01 / 0.022	[ −0.051 to 0.031 ] / [ −0.002 to 0.046 ]	0.618 / 0.071
NCEP	0.003 / -0.035	[ −0.047 to 0.052 ] / [ −0.087 to 0.017 ]	0.921 / 0.184
BNF	−0.033 / 0.005	[ −0.079 to 0.012 ] / [ −0.028 to 0.038 ]	0.151 / 0.777
ASSIGN	−0.007 / -0.016	[ −0.018 to 0.003 ] / [ −0.027 to −0.005 ]	0.177 / **0.003**
PROCAM	−0.033 / -0.328	[ −0.538 to 0.471 ] / [ −0.906 to 0.25 ]	0.897 / 0.264
Reynolds	−0.083 / 0.041	[ −0.165 to −0.002 ] / [ 0.009 to 0.073 ]	**0.044** / **0.013**
MESA	−0.005 / 0.154	[ −0.051 to 0.04 ] / [ 0.088 to 0.22 ]	0.817 / **<0.001**
ASCVD	0.01 / 0.001	[ −0.026 to 0.047 ] / [ −0.04 to 0.042 ]	0.579 / 0.957

*Bold indicates p < 0.05.

^†^Data represented as regression coefficient for women/men.

^‡^β = regression coefficient expressed as ms/per unit % change in risk score.

For women, only the MESA and Reynolds risk scores were significantly associated with both T_1_ time and ECV CMR metrics. Both MESA and Reynolds risk score showed inverse associations with post-contrast T_1_ time (i.e., greater fibrosis) in men (p = 0.008 and <0.001, respectively). ECV was positively associated with both MESA and Reynolds risk score (p = 0.001 and p = 0.013, respectively).

Most of the risk scores showed low to moderate changes in T_1_ time or ECV per unit change in risk percentage. Nonlinear GAM models resulted in improved correlation between the Reynolds risk score and 25 minute T_1_ time in men (p = 0.011). Similarly, non-linear association was observed for MESA risk score with 25 minute T_1_ time in men (p = 0.009). Table 9 summarizes these findings. Overall, these adjustments magnify the strength of correlations between either T_1_ times or ECV with risk scores below a predefined threshold determined using GAM analysis.

**Table 9:** **Relationship between CMR indices of fibrosis (T**_**1**_ **, ECV) and risk score models based on linear piecewise regression among men**

**Risk model**	**CMR index**	**β1**	**CI 1 (95%)**	**p-value**	**β2**	**CI 2 (95%)**	**p-value**
ASCVD	Native T_1_*	0.941	[ −0.266 to 2.148 ]	0.123	0.398	[ −0.033 to 0.830 ]	0.070
Reynolds	Post T_1_ (12 min)	−0.241	[ −0.721 to 0.239 ]	0.322	−0.419	[ −0.838 to −0.001 ]	**0.050**
Reynolds	Post T_1_ (25 min)	−0.498	[ −0.879 to −0.116 ]	**0.011**	−0.807	[ −1.829 to 0.214 ]	0.113
MESA	Post T_1_ (25 min)	−1.152	[ −2.012 to −0.292 ]	**0.009**	−0.504	[ −1.590 to 0.582 ]	0.360

*T_1_ time: 25 min post gadolinium T_1_ time.

β1, β2 for T_1_ time: shown as msec/%.

Bold indicates p-value ≤ 0.05

## Discussion

Risk scores predicting endpoints for men and women without symptomatic CVD are an increasingly important tool to help identify individuals who may benefit from more intensive or earlier medical intervention. Most risk models use similar risk factors to predict cardiovascular events (Table 2) and thus these models may be expected to show similar trends in relationship to CMR fibrosis. Greater CVD risk by most risk scores (10/14, 71%) was associated with greater myocardial fibrosis (25 min T1 time) as identified by CMR in men (Figure 1). T1 times (25 min) showed improved relationship to CVD risk scores, compared to other CMR measures such as ECV, native and 12 min T1 time. For women, who are generally categorized as lower risk than men in CVD models, there was generally little or no relationship between CVD risk score and T1 time or ECV.

Risk scores generally have similar endpoint prediction encompassing the likelihood of having coronary artery disease (CAD) that in turn may lead to heart attack and/or heart failure in late stage disease. We hypothesized that this propensity for cardiovascular disease may in turn be related to the development of diffuse myocardial fibrosis. Several previous studies have looked at the agreement amongst various risk scores, including those ones used for the current study. This study sought to indirectly look at agreement between any two risk scores based on their respective effect size and significance of associations with CMR indices. This agreement test assures a consistent comparison between risk scores. As in our study (Table 3), there was low agreement between any two of these risk scores as been reported, presumably due to the population dependence of these risk score estimates. However, each risk score has its own utilization scheme for its targeted population. Blending multiple risk scores together to have a single measure of risk for all subjects will unnecessarily de-emphasize the importance of some risk factors. Thus, we sought to evaluate a wide range of risk score methods in relationship to CMR indices of fibrosis.

CMR measures of diffuse myocardial fibrosis are relatively new, and long term validation in relationship to cardiovascular outcomes in a general population has not been performed. At present, there is little consensus regarding the ‘optimum’ CMR measure to quantify the degree of fibrosis especially in asymptomatic subjects. The partition coefficient is the slope of the linear relationship between myocardium relaxivity (1/ T_1_) vs. blood relaxivity before and after gadolinium administration [28]. The myocardial volume of distribution of gadolinium, or extracellular volume fraction (ECV), is derived from the partition coefficient by dividing by (1-hematocrit). ECV and partition coefficient seek to reflect the underlying correlates of diffuse myocardial fibrosis, i.e. expansion of the extracellular matrix. There are however multiple T_1_ measurements in the ECV calculation, each with its own error/variability in measurement. In contrast, change in T_1_ time is nonspecific as to etiology and does not entirely reflect myocardial fibrosis. Indeed, increased native T1 time and ECV have been reported in amyloid light chain disease and low T_1_ times have been reported in Fabry disease [29,30].

Our results were somewhat surprising, in that a ‘normalized’ T1 measure such as ECV might have been expected to perform better in relationship to risk scores compared to the 25 minute or other T1 measures. The exact reasons for this are primarily conjectural. Certainly most information about T1 measures to date derives from patients with clinical cardiovascular disease. For asymptomatic subjects, CVD risk factors such as hypertension (~50% of our study cohort), smoking, diabetes and age are probably those which may be most strongly related to myocardial fibrosis [9-12]. Myocardial fibrosis is a result of a pathologic process that involves extracellular matrix remodeling due to a wide array of causes: inflammation, excessive myocardial stretch, oxidative stress, and glycation products particularly from diabetic individuals [31]. Over time, the presence of myocardial fibrosis alters the myocyte arrangement in cardiac cells, thereby degrading the structure and function of the heart. Therefore, the most notable CVD risk factors are physiologically in tandem with the underlying pathology of myocardial fibrosis.

Of several possibilities, it is possible that a greater post gadolinium delay time (i.e. at 25 minutes) discriminates fibrosis and is relatively more reproducible in a multi-center setting, and thus more reliably related to cardiovascular risk factors compared to other T1 times. Nevertheless, it seems reasonable that CMR investigators continue to consider multiple T1 indices and that the optimal T1 index may vary by disease and/or condition being evaluated.

Since CVD risk models have been validated in various cohorts, it is reasonable to question if individuals deemed at higher CVD risk may have greater fibrosis indices by CMR. A critical factor in this analysis is whether CMR is sensitive enough to evaluate early myocardial fibrosis that might be predicted using these CVD risk calculators. Indeed, in men, this does appear to be the case: 10/14 CVD risk scores showed lower post-contrast T_1_ time in relationship to greater CVD risk. Of interest, ECV appeared to be less sensitive than post gadolinium T_1_ time in this regard. In the same fashion, native T_1_ time was also less sensitive than post-gadolinium T_1_ time. In women, little or no relationship was noted using the CMR indices. One reason for this may be substantially lower levels of both CVD risk and thus lower degrees of myocardial fibrosis in women compared to men.

The newly released risk score ASCVD 2013 showed poor correlation with T_1_ time and ECV for both men and women. Relative to the older risk scores, this new risk score reclassified substantial number of MESA subjects as “high risk”. This reclassification to high risk levels has been a major criticism of the new ASCVD risk score. Based on our CMR data, the new ASCVD score was not associated with CMR detected myocardial fibrosis.

Although independent of CMR fibrosis markers, the MESA risk score is derived from the same cohort as our study. Thus, it is not surprising that this score offers the greatest correlation between risk score and CMR indices of fibrosis. In addition, the MESA score adds more specific clinical markers to the risk score, including NT-proBNP levels and LV mass index. NT-proBNP is a strong independent predictor of outcomes in outpatients with chronic heart failure. Furthermore, diffuse myocardial fibrosis coincides with the processes leading up to an event of heart failure, indicative of progression of T_1_ times and ECV. LV mass index characterizes the structure of the heart, also reflective of the degree of diffuse fibrosis present. Potluri et al. previously have shown the inverse relationship between LV mass and T_1_ changes in more overt diseased cases such as HCM [32].

Like MESA, the Reynolds risk score is a contemporary risk score. The Reynolds risk score includes C reactive protein (CRP) in addition to traditional risk factors. CRP is a nonspecific marker for inflammation, but has been used to risk stratify subjects for cardiovascular disease. Furthermore, Zhang et al. has shown CRP promotes expression of angiotensin II-induced collagen type I and type III in mice [33], reflected by an increase in mRNA expression of collagen I. Because of this indirect effect of CRP on myocardial fibrosis, this risk factor may in some way contribute to the consistent strong correlations between Reynolds and CMR indices.

### Limitations

A limitation of the study is that the MESA subjects were asymptomatic healthy subjects at baseline and thus at relatively low cardiovascular risk. Nevertheless, 49% of men and 55% of women had hypertension, and 14% had diabetes. However a low prevalence of disease in asymptomatic individuals likely results in considerable error and thus variation in the risk prediction models (Table 3). In addition, these results are primarily correlative, which may lack causal interpretations in this study. The risk models all generally take into account age, gender, smoking, diabetes, blood pressure, cholesterol levels. Thus our results are limited to the conclusion that 25 minute T1 times are most strongly related to combinations of these risk factors. Other CMR measures (such as ECV) may be useful indicators of fibrosis in other, more definitive or more advanced disease states.

## Conclusion

Cardiovascular risk scores are routinely implemented to help identify patients who are at risk for subsequent cardiovascular events. The risk factors that comprise the risk scores have also been implicated in the development of myocardial fibrosis. Using CMR, diffuse myocardial fibrosis can be quantified, and greater CMR fibrosis correlated with greater CVD risk in men. These relationships were most prominent in the more contemporary risk scores derived from MESA and the Reynolds study using 25 minute T1 gadolinium time as an outcome measure. These moderate agreements between risk scores and T_1_ times suggest the clinical potential for CMR indices of fibrosis to be used in complement with risk scores, thereby adding prognostic value to patient care. However, future studies relating T_1_ time or ECV measurements to cardiovascular events will help to further refine the role of T1 mapping by CMR in asymptomatic individuals.

## Appendix

References and Links for CVD Risk Calculators

1. Edinburgh (CVD and CHD), BNF, ASSIGN, Stroke, CHD Death, CVD Death
  Payne R. Cardiovascular Risk Calculators [Internet]. Edinburgh, UK: University of Edinburgh; 2005 [cited 2014 Jan 15]. Available from: http://cvrisk.mvm.ed.ac.uk/calculator/calc.asp.
2. PROCAM Health Check
  PROCAM Health Check [Internet]. Switzerland: International Task Force for Prevention of Coronary Heart Disease; 2010 [cited 2014 Jan 15]. Available from: http://www.chd-taskforce.de/procam_interactive.html.
3. Framingham Heart Study (CVD)
  D'Agostino RB. et al. A General Cardiovascular Risk Profile for Use in Primary Care: The Framingham Heart Study [Internet]. US: The Framingham Heart Study; 2008 [cited 2014 Jan 15]. Available from: https://www.framinghamheartstudy.org/risk-functions/cardiovascular-disease/10-year-risk.php.
4. National Cholesterol Education Program
  Grundy SM, et al. Risk Assessment Tool for Estimating Your 10-year of Having a Heart Attack [Internet]. US: Third Report of the Expert Panel on Detection, Evaluation, and Treatment of High Blood Cholesterol in Adults (Adult Treatment Panel III); 2001 [cited 2014 Jan 15]. Available from: http://hp2010.nhlbihin.net/atpiii/calculator.asp.
5. Reynolds Risk Score
  Ridker PM, et al. Reynolds Risk Score [Internet]. US: Reynoldsriskscore.org; 2007 [cited 2014 Jan 15]. Available from: http://www.reynoldsriskscore.org/Default.aspx.
6. MESA Heart Failure
  Chahal, Harjit et al. Traditional and Novel Risk Factors can Estimate Five Year Risk of Developing Heart Failure: Results from the Multi-ethnic Study of Atherosclerosis [cited 2014 Jan 15]. Available from: http://circ.ahajournals.org/cgi/content/meeting_abstract/126/21_MeetingAbstracts/A11145.
7. ASCVD
  Goff, D.C., Jr., et al., 2013 ACC/AHA Guideline on the Assessment of Cardiovascular Risk: A Report of the American College of Cardiology/American Heart Association Task Force on Practice Guidelines. Circulation, 2013 [cited 2014 Jan 15].
